# False memory in between-language conditions: a brief review on the effect of encoding and retrieving in different languages

**DOI:** 10.3389/fpsyg.2023.1237471

**Published:** 2023-08-10

**Authors:** Maria Soledad Beato, Mar Suarez, Sara Cadavid, Pedro B. Albuquerque

**Affiliations:** ^1^Faculty of Psychology, University of Salamanca, Salamanca, Spain; ^2^School of Medicine and Health Sciences, Universidad del Rosario, Bogotá, Colombia; ^3^School of Psychology, University of Minho, Braga, Portugal

**Keywords:** second language, false recognition, DRM paradigm, between-language false memory, encoding, retrieval

## Abstract

False memories have been extensively investigated over the past few decades using the Deese/Roediger-McDermott (DRM) paradigm. In this paradigm, participants study lists of words associatively related to a non-presented critical lure. During a memory test, these critical lures are falsely recalled or recognized. Most studies have focused on false memories that arise when both encoding and retrieval are conducted in the same language (i.e., within-language conditions), which is typically the participant’s native or first language (L1). However, much less is known about false memories when critical lures appear in the memory test in a different language than the studied lists (i.e., between-language conditions), being one of them the participant’s second language (L2). The main objective of this exhaustive review was to provide an overview of the current state of research on false recognition using the DRM paradigm in between-language conditions, where languages are switched between encoding and retrieval (i.e., L1L2 versus L2L1). The results revealed a language dominance effect in between-language false memories. In other words, false recognition rates were dependent on the study language, with a trend toward higher false recognition when words were enconded in the L1 (L1L2) compared to when words were encoded in the L2 (L2L1). This review enhances our understanding of how studying words in a first or second language affects false memory in the DRM paradigm, emphasizing the significance of investigating false memory in second language speakers and the necessity for further research in the field.

## 1. Introduction

Memory researchers worldwide have been captivated by the robustness of the false memory effect using the Deese/Roediger-McDermott (DRM) paradigm (Deese, 1959; Roediger and McDermott, 1995), one of the most used techniques to study false memories. In this paradigm, participants are presented with lists of words (e.g., fountain, bridge, pool, boat, swim, fish) associated with a non-presented critical lure (e.g., WATER). In a subsequent memory test, participants frequently falsely recall or recognize the critical lures as studied items. An extensive literature suggests that false memories are a universal phenomenon that can occur across different languages and cultures (e.g., Anaki et al., 2005; Chen et al., 2008; Diekelmann et al., 2010; Dubuisson et al., 2012; Carneiro et al., 2014; Cadavid et al., 2021; Beato et al., 2023b). The consistency of this effect across diverse languages presents an excellent opportunity to contribute to the existing literature concerning the influence of linguistic context on memory (e.g., Marian and Kaushanskaya, 2007; Kroll et al., 2010; Ning et al., 2020).

Although the study of false memories has primarily centered on monolinguals’ native language, investigating them in a second language has emerged as an important field of research, particularly in today’s globalized world where bilingualism is increasingly prevalent. As more individuals acquire and use a second language, understanding how this linguistic context affects memory, including false memory, holds significant importance. Therefore, to fully understand false memories, researchers should examine their occurrence in both the participants’ first language (hereafter L1) and second language (hereafter L2). Unfortunately, limited research has been conducted on false memory in a non-dominant language (e.g., Anastasi et al., 2005; Howe et al., 2008; Marmolejo et al., 2009; Suarez and Beato, 2023), leading to a lack of understanding in this area.

At a theoretical level, bilingual research explores the representation of two languages in the brain. Various models have been developed to address this question, including bilingual interactive activation model (Dijkstra and Van Heuven, 2002), distributed feature model (de Groot, 1992), revised hierarchical model (Kroll and Stewart, 1994; Kroll et al., 2010), ontogenesis model of L2 lexical representation (Bordag et al., 2022), among others. Despite differences in L1 and L2 representations, these models share two key assumptions: a shared conceptual system accessed by both languages (Francis, 1999, 2020), and stronger associations between word forms and concepts in L1 compared to L2 (e.g., Gollan et al., 2008). In terms of false memories, they have traditionally been explained by activation-monitoring framework (Roediger et al., 2001) or fuzzy-trace theory (Brainerd and Reyna, 2002). To provide clarity to our results, this study is grounded in the revised hierarchical model (RHM) from bilingual research and the activation-monitoring framework (AMF) from the false memory literature, as they both offer predictions related to activation processes. This approach, used in previous research (e.g., Suarez and Beato, 2023; Beato et al., 2023a), enables an integrated discussion of our findings.

Based on these two teories, false memories in the DRM paradigm are expected to be higher when words are studied in L1 than in L2. The RHM proposes stronger conceptual links in the dominant language (L1) compared to the non-dominant language (L2), except when speakers have similar proficiency in both languages. Therefore, the RHM predicts a stronger activation of concepts from L1 than L2 words. Once the concept of the studied words has been activated, the AMF suggests that this activation spreads throughout a well-organized network with stronger connections to associatively related words (i.e., critical lure) in the L1 than in the L2, leading to higher false memories when words are studied in the dominant than the non-dominant language. A prior literature review conducted by Suarez and Beato (2021) supports this assumption. The authors examined the available studies on false recognition in the dominant language (L1) compared to the non-dominant language (L2) in within-language conditions, focusing on L2 proficiency. They concluded that speakers with higher proficiency in their L1 than their L2 had significantly more false memories in their dominant language (L1 > L2), known as the language dominance effect.

While the results regarding within-language false memories in both L1 and L2 are well-established, there is a lack of comprehensive reviews exploring between-language false memories (i.e., memory distortions that occur in one language after encoding words in another language). The occurrence of between-language false memories, where individuals falsely retrieve a non-presented critical lure in a different language than their studied associates, provides evidence that false memories are triggered by automatic and spontaneous processes that raise associative distortions (Otgaar et al., 2017). Hence, studying between-language false memory constitutes a privileged window into the fundamental mechanisms of false memory formation and the intrincate relationship between language context and memory.

The purpose of this exhaustive review was to analyze the current state of research on false memory in between-language conditions, in which languages are switched between encoding and retrieval (i.e., L1L2 and L2L1). By conducting a thorough literature search and analysis of the available articles investigating this topic, we aimed to better understand the impact of study language on between-language false recognition. The analysis of relevant articles provides insights into the factors influencing false memories in between-language conditions, shedding light on the complex dynamics inherent in false memory processes, particularly among second language speakers.

## 2. False memory in between-language conditions: L1L2 and L2L1

Previous studies investigating false memory in different languages using the DRM paradigm have employed various types of comparisons. A common analysis compares false recognition between conditions where the study and test languages matched (L1L1 or L2L2) and did not match (L1L2 or L2L1), that is, within- and between-language conditions, respectively. Moreover, these studies have used two types of memory instructions (for more information, see Beato et al., 2023a): restrictive instructions require participants to retrieve language information to confirm whether the study and test languages match, while inclusive instructions instruct participants to endorse studied words regardless of language match between study and test.

Beato et al. (2023a) compared within and between-language conditions following restrictive and inclusive instructions (Experiment 1 and 2, respectively). With restrictive instructions, false recognition was greater in within-language conditions than in between-language conditions. This pattern was consistent for both L1 (L1L1 > L1L2) and L2 (L2L2 > L2L1) studied words, replicating previous findings (Cabeza and Lennartson, 2005; Sahlin et al., 2005). Essentially, participants were able to retrieve the study language during the memory test, and if it did not match the test language, they rejected the items, leading to lower false recognition in between-language than within-language conditions. With inclusive instructions, false recognition was also higher in within- than between-language conditions, but only when the study language was the L1.

The latter finding, together with the effect of language dominance mentioned earlier (e.g., Anastasi et al., 2005; Arndt and Beato, 2017; for a review, see Suarez and Beato, 2021), shows that the automaticity level (i.e., how fast, strong, and more readily concepts are being activated) of the encoding processes is critical to raise false memories. Since the encoding phase seems to be crucial for false memory formation, in this review, we expected to find the effect of language dominance in between-language conditions as well. This means that when there is a mismatch between the study and test languages, and participants differ in the proficiency of the two languages, between-language false recognition would be expected to be higher when words were encoded in the L1 than in the L2 (L1L2 > L2L1).

To test this hypothesis, we will explore the limited number of studies on between-language false memory using the DRM paradigm. Despite its importance in understanding memory processes, research on the effects of language mismatch on false memory remains relatively unexplored. To our knowledge, only six studies (Table 1) have explored the effect of language mismatch on false memory (Kawasaki-Miyaji et al., 2003; Cabeza and Lennartson, 2005; Sahlin et al., 2005; Howe et al., 2008; Marmolejo et al., 2009; Beato et al., 2023a). Not only are there few studies, but also one of these studies, Howe et al. (2008), did not directly compare between-language conditions, as these conditions were collapsed across all analyses. Therefore, specific information on the comparison among between-language conditions, which is the primary focus of this review, is lacking in this study.

**Table 1:** Summary of the reviewed studies analyzing between-language false recognition.

Authors, year		Languages		Participants		Language proficiency and background	Memory instructions	Results: false recognition		
		L1	L2	No.	Age (*M*)			L1L2	L2L1	Conclusion
Kawasaki-Miyaji et al. (2003)		Japanese	English	74	University students (N/A)	L1: dominant language L2: 7 years of academic training Participants lived in Japan	Restrictive recognition	0.65^1^	0.69^1^	L1L2 ≈ L2L1^2^
Cabeza and Lennartson (2005)		English (for 80%)	French	30	University students (N/A)	L1 and L2: high proficiency and used in everyday life Participants lived in Edmonton, Canada (English-speaking environment)	Restrictive recognition	0.39	0.25	L1L2 > L2L1
Sahlin et al. (2005)		English	Spanish	20	University students (20.00)	L1 (dominant language): proficiency self-report = 5/5 L2: proficiency self-report = 4.55/5 Participants lived in the U.S.	Restrictive recognition	0.38	0.33	L1L2 > L2L1^2^ (data from the first trial)
Howe et al. (2008)		English	French	160 128 120 80	6 years old 8 years old 12 years old 20 years old	L1 (children and adults): dominant language L2 (children): L2-immersion school (only L2-speaking environment) L2 (adults): proficient in French according to a test and work environment All lived in an L1 community in Canada	Inclusive recognition	**—**	**—**	No comparisons among between-language conditions
Marmolejo et al. (2009)		English	Spanish	119	University students (20.63)	L1 (dominant language): proficiency self-report = 9.35/10 L2: proficiency self-report = 8.40/10 Participants lived in the U.S.	Inclusive recognition	0.87	0.80	L1L2 > L2L1^2^
Beato et al. (2023a)	Exp 1	Spanish	English	90	University students (20.61)	L1: dominant language L2: studied in primary and secondary school. Proficiency self-report = 5.68/10 Participants lived in Spain	Restrictive recognition	0.15	0.15	L1L2 ≈ L2L1
	Exp 2	Spanish	English	90	University students (20.24)	L1: dominant language L2: studied in primary and secondary school. Proficiency self-report = 4.94/10 Participants lived in Spain	Inclusive recognition	0.48	0.39	L1L2 > L2L1

1 Means were provided by the first author in Kawasaki-Miyaji et al. (2003).

2 The comparison was not tested statistically.

The first published study using DRM lists in different languages involved balanced and unbalanced Japanese-English bilinguals (Kawasaki-Miyaji et al., 2003). Seventy-four Japanese undergraduates studied six 15-word lists in Japanese (L1) and six in English (L2). The memory test was a 4-alternative forced-choice test, where participants had to indicate whether each word was: (1) presented in English, (2) presented in Japanese, (3) presented but unsure about the language, or (4) not presented. It included 36 studied items, 18 presented in the same language as the encoding phase (i.e., within-language condition) and 18 in the other language (i.e., between-language condition). L2 critical lures were presented in either the same or the other language as their list in the study phase. Additionally, 24 unrelated distractors were presented (half in the L1, half in the L2). Results showed similar false recognition in both between-language conditions, although this difference was not tested for statistical significance.

Focusing on research that included participants with high proficiency in both L1 and L2, in Cabeza and Lennartson’s (2005) study participants studied 10 DRM lists in English and 10 in French. The recognition test included 20 distractors, 20 studied words, and 20 critical lures, half presented in English and half in French to either match or not the language of study. Participants were instructed to recognize as “old” only the words presented in the same language during the study and test phases (i.e., restrictive memory instructions). Results showed that in between-language conditions false recognition was higher in L1L2 (*M* = 0.39) than L2L1 condition (*M* = 0.25), indicating increased false memory when studying in the L1.

Sahlin et al. (2005) also studied highly proficient bilinguals. They recruited 20 English-Spanish undergraduates living and studying in an English-speaking environment. Given their slightly better performance in English than Spanish, English was considered their dominant language (L1) and Spanish their non-dominant language (L2). Participants studied 12 10-word lists (six in each language) and underwent a yes/no recognition test. This study-test procedure was repeated five times, but for the sake of comparability with the other studies, we will focus on discussing the results of the first study-test cycle. For each of the 96 words of the recognition test, participants responded whether the word was studied in the same language (i.e., restrictive memory instructions). The test included 36 studied words, 12 critical lures, 36 unrelated distractors, and 12 unrelated-critical distractors, for which half were presented in English and half in Spanish. To established within- and between-language conditions, six lists studied in one language were tested in the same language, and the other six were tested in the other language. Regarding the primary interest of this review, comparing L1L2 and L2L1, the corrected proportions of false recognition in the first trial suggested a clear trend: false recognition was higher in L1L2 than in L2L1 (0.32 vs. 0.25, respectively), although this was not statistically tested.

Marmolejo et al. (2009) conducted a study with highly proficienct English-Spanish bilinguals. Participants encoded ten 12-word DRM lists in English or in Spanish. Then, they underwent a recall test, with half of the lists being retrieved in English and half in Spanish, and a final 60-word yes/no recognition test (including 30 studied words, 10 critical lures, 15 unrelated distractors, and five unrelated-critical distractors) presented either in English or in Spanish, followed by a ± 3 confidence rating. Participants should endorse the words that were previously presented, regardless of the study language (i.e., inclusive memory instructions). Regarding the comparison of interest, as expected, between-language false recognition was higher in L1L2 than L2L1 (0.87 vs. 0.80, respectively), although this difference was not tested for statistical significance. Hence, Marmolejo et al.’s (2009) study also showed a trend of higher false recognition in L1L2 than L2L1, similar to previous studies.

This trend was also found in Beato et al. (2023a), but only in one of their experiments. The study included restrictive and inclusive memory instructions (Experiment 1 and 2, respectively). Ninety undergraduates studied 16 10-word DRM lists (8 in Spanish, L1; 8 in English, L2). The recognition test included 96 words: 48 studied words presented in the same language as the study phase (24 in L1, 24 in L2), and 48 non-studied words (32 unrelated distractors and 16 critical lures). Half of the critical lures were presented in the same language as their study lists and half were translated into the other language. Comparing L1L2 and L2L1 conditions, Beato et al. (2023a) found higher false recognition in L1L2 compared to L2L1 (0.48 vs. 0.39, respectively), but only with inclusive instructions (Experiment 2). No significant difference was found with restrictive instructions (Experiment 1).

## 3. Discussion

This review examines the current state of research on false memory in between-language conditions (L1L2 and L2L1), where languages are switched between encoding and retrieval. It aims to gain a deeper understanding of between-language false recognition and uncover the intricate dynamics of false memory processes, particularly among second-language-speaking populations. Despite the limited number of studies and inconsistency of the findings, this review sought to fill the knowledge gap by offering a comprehensive analysis of the available studies.

We anticipated to find an effect of language dominance in between-language conditions, particularly when participants differ in their L1 and L2 proficiency. That is, when there is a mismatch between the study and test languages, it was expected that between-language false recognition would be higher when words were encoded in the L1 (L1L2) than in the L2 (L2L1). All the studies included in this review recruited participants with a language that was dominant over the other (L1 vs. L2). As a result, it was expected that these participants would exhibit faster and more automatic activation of conceptual representations from words in their L1 compared to their L2 (Kroll and Stewart, 1994; Kroll et al., 2010). Consequently, they would produce more false recognition under L1L2 than L2L1 conditions.

As expected, most of the studies reported a trend of higher false recognition in the L1L2 than the L2L1 conditions (Cabeza and Lennartson, 2005; Sahlin et al., 2005; Marmolejo et al., 2009; Beato et al., 2023a, Exp. 2), indicating an effect of language dominance in between-language false memory. However, other studies did not find significant differences when comparing between-language conditions (Kawasaki-Miyaji et al., 2003; Beato et al., 2023a, Exp. 1). A detailed examination of these two experiments showed that they both have some peculiarities that could explain why they did not find the language dominance effect.

There are at least two reasons why the Kawasaki-Miyaji et al.’s (2003) study is not comparable to the other studies. First, participants responded to a recognition test that was itself a source-monitoring test. This memory test may have implied that more cognitive resources were allocated to the decision-making processes for each item. Having to carry out such strategic processes may have made the decision for each word less dependent on automatic activation. Hence, Kawasaki-Miyaji et al.’s recognition test might have led the error-editing processes to overshadow the language dominance effect. Second, and more notably, in this study, the L1 and the L2 were encoded and tested in different scripts. On the one hand, Japanese uses one logographic (i.e., Kanji) and two phonological scripts (i.e., Hiragana and Katakana). On the other hand, English uses just a phonological script that resorts to an alphabetic orthography. There is a vast amount of research on the script switching cost, showing that there are delays in reading and semantic categorization (e.g., Dylman and Kikutani, 2018), which could affect memory processes. Therefore, Kawasaki-Miyaji et al.’s (2003) results are worth comparing to other cross-script studies that have not been done so far.

For its part, Experiment 1 by Beato et al. (2023a) stands out from other studies due to its unique pattern of results and the inclusion of participants with low-L2 proficiency. This experiment employed restrictive instructions, which require participants to focus on the lexical representation of the test word rather than just the concept. That is, even if they have a false memory of the concept, they would have to reject it if they think the word presented is a translation of the previously studied one. The RHM proposes that participants with low proficiency in the L2 show much stronger conceptual links in L1 than L2 (Kroll and Stewart, 1994; Kroll et al., 2010). Consequently, when encountering words in the L2, they do not access the concept directly and quickly, but access it via the translation into the L1. Therefore, low-proficiency participants in the L1L2 condition are expected to reach activation of the critical lure in the L1. The L2 concept word is not likely to be activated in this condition because the connections between L1 and L2 and from the concept itself to the L2 are weak in low-proficiency participants. Hence, if presented at test with the critical lure translated into the L2, participants would reject it, producing very little false recognition in the L1L2 condition. For its part, in the L2L1 condition, critical lures are expected to receive very limited activation since the words are encoded in a non-dominant language in which people do not have much competence. In conclusion, when using restrictive memory instructions with low-proficiency participants, one would expect a reduction in false recognition that would be similar for L1L2 and L2L1 conditions. This is precisely what Beato et al. (2023a) found in their first experiment.

In summary, the findings showed a higher false recognition in L1L2 than L2L1 conditions. This highlights the importance of considering the encoding language when studying false memories. In other words, the language in which the list items were initially encoded was found to be a crucial factor influencing false recognition, rather than the language of the critical lure during retrieval, emphasizing that language is not a separate module but a critical factor influencing memory processes. Therefore, this review sheds light on the relationship between language and memory, making valuable contributions to the field of bilingual cognition, and aligns with theoretical the models mentioned in the introduction (e.g., Kroll and Stewart, 1994; Roediger et al., 2001; Kroll et al., 2010), proposing stronger associations between words and concepts in the dominant language (L1) compared to the non-dominant language (L2). Additionally, these findings enhance our understanding of the intricate dynamics of false memory formation in different language contexts.

Finally, the limited number of studies exploring between-language false memory using the DRM paradigm hinders our understanding of this complex phenomenon. Further research is necessary to gain a comprehensive understanding of false memory processes in between-language conditions. Additionally, while most reviewed studies suggest a language dominance effect in between-language false recognition, it is important to note that some data trends were not statistically analyzed for the intended comparison. Thus, more research is needed to fully comprehend the factors influencing false memories in between-language conditions and the underlying mechanisms of false memory formation in second language speakers.

## Author contributions

MSB: conceptualization, writing original draft, supervision, and formal analyses. MS: methodology, writing – review and editing, and formal analyses. SC: methodology, writing – review and editing, and formal analyses. PBA: writing – review and editing.

## Funding

This study was partially supported by the University of Salamanca, and the Foundation for Science and Technology (FCT) through the Portuguese State Budget (UIDB/01662/2020).

## Conflict of interest

The authors declare that the research was conducted in the absence of any commercial or financial relationships that could be construed as a potential conflict of interest.

## Publisher’s note

All claims expressed in this article are solely those of the authors and do not necessarily represent those of their affiliated organizations, or those of the publisher, the editors and the reviewers. Any product that may be evaluated in this article, or claim that may be made by its manufacturer, is not guaranteed or endorsed by the publisher.
